# Ratio between Negative and Positive Lymph Nodes Is Suitable for Evaluation the Prognosis of Gastric Cancer Patients with Positive Node Metastasis

**DOI:** 10.1371/journal.pone.0043925

**Published:** 2012-08-31

**Authors:** Jingyu Deng, Dan Sun, Yuan Pan, Li Zhang, Rupeng Zhang, Dianchang Wang, Xishan Hao, Han Liang

**Affiliations:** 1 Gastric Cancer Surgery Division, Tianjin Medical University Cancer Institute and Hospital, Key Laboratory of Cancer Prevention and Therapy, Tianjin, China; 2 Institute of Lung Cancer, General Hospital of Tianjin Medical University, Tianjin, China; 3 Hepatobilliary Cancer Surgery Division, Tianjin Medical University Cancer Institute and Hospital, Key Laboratory of Cancer Prevention and Therapy, Tianjin, China; University of North Carolina School of Medicine, United States of America

## Abstract

**Objective:**

To date, there is no consensus to evaluate the most appropriate category of the nodal metastasis for precise predication the prognosis of gastric cancer patients with positive node metastasis after curative surgery.

**Methods:**

We retrospectively analyzed the clinicopathologic characteristics and overall survival (OS) of 299 gastric cancer patients with positive node metastasis after curative surgery for evaluation the optimal category of the nodal metastasis.

**Results:**

With the univariate and multivariate survival analyses, the depth of primary tumor invasion was identified as the independent predicators with the OS of 299 gastric cancer patients with nodal metastasis postoperatively, as were the number of positive lymph nodes (PLNs), the number of negative lymph nodes (NLNs), and the ratio between negative and positive lymph nodes (RNPL). The RNPL was identified to be more suitable for predication the OS of gastric cancer patients with positive node metastasis than the ratio between positive and dissected lymph nodes (RPDL) by using the stratum procedure of survival analysis. Besides, we found both PLNs and NLNs were independently correlated with OS of gastric cancer patients with nodal metastasis when RNPL, instead of RPDL, was controlled in the partial correlation model.

**Conclusions:**

RNPL, a new category of the nodal metastasis, was suitable for predication the OS of gastric cancer patients with nodal metastasis after curative resection, as were the PLNs, and NLNs.

## Introduction

Presence of lymph node metastasis from primary tumor is one of most important indicator for predication the prognosis of gastric cancer postoperatively, as is depth of tumor invasion [1], [2]. However, it is not the consensus of the optimal category of the nodal metastasis for predication the prognosis of gastric cancer patients after surgery worldwide. The Japanese category of the nodal metastasis (n stage) for evaluation the prognosis of gastric cancer patients based on the anatomical location of positive nodes was first proposed by the Japanese Gastric Cancer Association (JGCA) in the 1960s [3]. However, the category of the nodal metastasis based on the location of metastatic lymph nodes was found to be inferior to the category of the nodal metastasis based on the number of positive lymph nodes (PLNs) for predication the prognosis of gastric cancer, and was considered to be preferable for surgical instruction rather than prognostic predication [4], [5]. At present, the PLNs-based classification is considered an appropriate category of nodal metastasis in accordance with the N stage proposed by the International Union Contrele Cancer (UICC) and American Joint Commission for Cancer (AJCC) in 1997 [6], [7]. Whether or not the ratio between positive and dissected lymph nodes (RPDL) is superior to PLNs for use in the precise prediction of gastric cancer prognosis following radical surgery remains controversial [8]. Nevertheless, several investigators have reported that RPDL is the best category of the nodal metastasis for evaluation the postoperative OS of gastric cancer patients [9]–[11]. Our published article showed that PLNs are more effective than RPDL in determining the postoperative OS of gastric cancer patients [12].

Recently, we have reported that number of negative lymph nodes (NLNs) is an important predicator of the OS of gastric cancer patients after curative surgery in addition to the extent of lymph node metastasis, the PLNs, and the RPDL [13]. Further, we demonstrated the NLNs had positive associations with the OS of gastric cancer patients following the extended lymphadenectomy [14]. Consequently, we did take it for granted that NLNs should be regarded as a new category of the nodal metastasis for evaluation the postoperative prognosis of gastric cancer patients.

In view of aforementioned causalities, we designed this study to address several issues which were associated with nodal metastasis from gastric cancer. They are as follows: 1) to elucidate the suitable categories of the nodal metastasis for predication the OS of gastric cancer patients with positive node metastasis after curative resection; and 2) to initially interpret the superiorities and reasons of the suitable categories of the nodal metastasis for predication the OS of gastric cancer patients with positive node metastasis after curative resection.

## Methods

### Patients

1748 patients who underwent potentially curative resection for gastric cancer at the Gastric Cancer Surgery Division, Tianjin Medical University Cancer Hospital from January 1997 through December 2003 were eligible for this study. Eligibility criteria for this study included: 1) histologically proven primary adenocarcinoma of the stomach, 2) no history of gastrectomy or other malignancy, 3) a lack of non-curative surgical factors except for distant metastasis (such as liver, lung, brain, or bone-marrow metastasis) and peritoneal dissemination, lymph node metastasis in para-aortic lymph node metastasis, 4) lymphadenectomy performed (limited, or extended), 5) no gastroesophageal junction tumor or cardia tumor, 6) the number of dissected lymph nodes for pathological examination was no less than 15, 7) positive node metastasis identified by pathological examination postoperatively, and 8) no patients died during the initial hospital stay or for 1 month after surgery. As a result, 1449 patients were excluded from this study. Of these excluded patients, 31 had the history of gastrectomy, 52 had other malignancy, 43 presented with hepatic metastasis intra-operation, 63 had ovarian metastasis, 221 underwent palliative gastrectomy for para-aortic node metastasis, 106 had peritoneal dissemination, 34 died of serious complications, 742 had less than 15 dissected lymph nodes, and 157 identified pathologically to have no node metastasis. Ultimately, 299 patients were included in this study.

### Surgical Treatment

All patients were operated on according to the potentially curative gastrectomy plus lymphadenectomy method. Curative resection was defined as a complete lack of grossly visible tumor tissue and metastatic lymph nodes remaining after resection, with pathologically negative resection margins [15]. Primary tumors were resected en bloc with limited or extended lymphadenectomy (D1 or D2–3 according to the Japanese Gastric Cancer Association [16]). The choice of surgical procedure of gastrectomy (total gastrectomy or subtotal gastrectomy) was made by the attending surgeon’s preference, and based mainly on the gastric cancer treatment guidelines in Japan [17]. Surgical specimens were evaluated as recommended by 7th UICC TNM classification for gastric cancer.

### Adjuvant Therapy

Most of patients received the adjuvant chemotherapy based on fluorouracil and leucovorin calcium after curative gastrectomy. Radiotherapy was not routinely administrated in patients routinely.

### Evaluated Variables

To determine the most appropriate cut-off values for continuous data variables, such as age at surgey, tumor size, RPDL, TLNs, NLNs, and ratio between negative and positive lymph nodes (RNPL), the cut-point survival analysis [12], [18] was adopted. The following clinicopathological variables were evaluated: (1) age at surgery (<55, or ≥55 years); (2) gender (male or female); (3) tumor location (lower third, middle third, upper third, or whole stomach); (4) tumor size (≤6.5, or >6.5c m); (5) extent of lymphadenectomy (limited, or extended); (6) type of gastrectomy (subtotal, or total); (7) Lauren’s classification of primary tumor (intestinal, diffuse, or mixed); (8) depth of primary tumor invasion (according to 7th UICC TNM Classification T stage) (T1, T2, T3, or T4); (9) extent of lymph node metastasis (perigastric (n1 stage according JGCA), or extragastric (n2, or n3 stage according to JGCA); (10) TLNs (≤22, or >22); (11) PLNs (according to 7th UICC TNM Classification N stage) (N0, N1, N2, or N3); (12) NLNs (≤9, or >9); (13) RNPL (≤0.18, 0.19–1.70, 1.71–7.00, or >7.00); (14) RPDL (≤10.0%, 10.1–40.0%, and >40.0%); and (15) the 7th UICC TNM Classification (Ia, Ib, IIa, IIb, IIIa, IIIb, IIIc, and IV).

### Ethics Statement

The study was approved by the Research Ethics Committee of Tianjin Medical University Cancer Institute and Hospital, China. Informed consent was obtained from all patients before participating in the study.

### Statistical Analysis

Categorical variables were statistically compared a χ2 or Fisher’s exact test. Continuous data were shown as mean (s.d.) and were statistically compared using the Mann–Whitney test. The median OS was determined by using the Kaplan-Meier method, and log-rank test was used to determine significance. Factors that were deemed of potential importance on univariate analyses (*P*<0.05) were included in the multivariate analyses. Multivariate analysis of OS was performed by means of the Cox proportional hazards model, using the forward: Logistic regression (LR) procedure for variable selection, respectively. Hazard ratios (HR) and 95% CI were generated. Akaike Information Criterion (AIC) and Bayesian Information Criterion (BIC) were performed for evaluation the best clinicopathological variable for predication the prognosis of gastric cancer. The smaller are AIC value and BIC value, the better is clinicopathological variable for predication the prognosis. Bivariate correlation analysis performed by means of the bivariate correlation model for validation the correlation between the given variable associated with lymph node metastasis and the OS of patients. Partial correlation analysis among variables associated with lymph nodes metastasis was performed by means of the partial correlation model for validation whether the given variable had any impact on the correlation between the OS of patients and the other variable. In all statistical analyses, significance was defined as *P*<0.05 and the statistical significance was two sided. The OS analysis of all patients was initially completed in April 2009. All statistical analyses were performed with statistical analysis program package (SPSS 16.0, SPSS Inc. Chicago, IL).

### Follow-up

After curative surgery, all patients were followed every 6 months for 2 year, then every year or until death. The median follow-up for the entire cohort was 54 months (range: 3–127). The follow-up of all patients who were included in this study was completed in February 2009. B ultrasonography, CT scans, chest X-ray, and endoscopy were obtained with every visit.

**Table 1:** Clinicopathologic characteristics of 299 gastric cancer patients with positive node metastasis.

**Gender**			
Male	208 (69.6%)		
Female	91 (30.4%)		
**Age at surgery**			
	**Mean±SD:** 54.85±11.86 years **Range:** 22–78 years		
<55	151 (50.5%)		
≥55	148 (49.5%)		
**Tumor location**			
Lower third	151 (50.5%)		
Middle third	76 (25.4%)		
Upper third	67 (22.4%)		
More than 2/3	5 (1.7%)		
**Tumor size**			
	**Mean±SD:** 6.19±2.83 cm **Range:** 1.5–19.0 cm		
≤6.5	185 (61.9%)		
>6.5	114 (38.1%)		
**Depth of primary tumor invasion** (according to 7th UICC TNM Classification T stage)			
T2	2 (0.7%)		
T3	8 (2.7%)		
T4a	241 (80.6%)		
T4b	48 (16.1%)		
**Extent of lymph node metastasis**			
Perigastric	220 (73.6%)		
Extragastric	79 (26.4%)		
**TLNs**			
	**Mean±SD:** 21.97±8.15 **Range:** 15–64		
≤22	206 (68.9%)		
>22	93 (31.1%)		
**PLNs** (according to 7th UICC TNM Classification N stage)			
	**Mean±SD:** 8.80±8.00 **Range:** 1–53		
N1	83 (27.8%)		
N2	68 (22.7%)		
N3	148 (49.5%)		
**NLNs**			
	**Mean±SD:** 13.18±8.59 **Range:** 0–61		
≤9	98 (32.8%)		
>9	201 (67.2%)		
**RPDL**			
	**Mean±SD:** 39.69%±29.58% **Range:** 1.6%–100%		
≤10.0%	69 (23.1%)		
10.1%–40.0%	97 (32.4%)		
>40.0%	133 (44.5%)		
**RNPL**			
	**Mean±SD:** 5.57±8.00 **Range:** 0–61		
≤0.18	27 (9.0%)		
0.19–1.70	121 (40.5%)		
1.71–7.00	76 (25.4%)		
>7.00	75 (25.1%)		
**Type of gastrectomy**			
Subtotal	223 (74.6%)		
Total	76 (25.4%)		
**Extent of lymphadenectomy**			
Limited	105 (35.1%)		
Extended	194 (64.9%)		
**Lauren’s classification of primary tumor**			
Intestinal	145 (48.5%)		
Mixed	126 (42.1%)		
Diffuse	28 (9.4%)		
**TNM Classification** (the 7th edition)			
IIa	1 (0.3%)		
IIb	3 (1.0%)		
IIIa	79 (26.4%)		
IIIb	61 (20.4%)		
IIIc	155 (51.8%)		

SD, standard deviation.

## Results

### Clinicopathological Outcomes

The clinicopathological characteristics of 299 gastric cancer patients with positive nodes after curative resection are shown in Table 1. The 5 year survival rate (5-YSR) of all enrolled patients was 32.1%, and 84 patients were alive when the follow-up was completed. The median OS of all patients after surgery was 27.0 months.

### Univariate Survival Analysis

With univariate analysis (Kaplan-Meier method), we found twelve clinicopathological variables had significant associations with the OS of gastric cancer patients with positive node metastasis after curative resection. They are as follows: age at surgery, tumor location, tumor size, type of gastrectomy, Lauren’s classification of primary tumor, depth of primary tumor invasion, extent of lymph node metastasis, PLNs, NLNs, RPDL, RNPL, and the 7th UICC TNM Classification of gastric cancer (Table 2).

**Table 2:** Univariate survival analysis of 299 gastric cancer patients with positive node metastasis.

Variables	MedianOS (mo)	Chi-squarevalue	*P* value
**Gender**			
Male	25	0.448	0.503
Female	33		
**Age at surgery (years)**			
<55	36	10.467	**0.001**
≥55	25		
**Tumor location**			
Lower third	35	11.592	**0.009**
Middle third	16		
Upper third	26		
More than 2/3	36		
**Tumor size (cm)**			
≤6.5	35	12.998	**<0.001**
>6.5	22		
**Type of gastrectomy**			
Subtotal	34	21.117	**<0.001**
Total	14		
**Extent of lymphadenectomy**			
Limited	27	0.013	0.911
Extended	28		
**Lauren’s classification of primary tumor**			
Intestinal	35	9.275	**0.010**
Mixed	36		
Diffuse	24		
**Depth of primary tumor invasion** *			
T2	75	25.011	**<0.001**
T3	24		
T4a	33		
T4b	15		
**Extent of lymph node metastasis**			
Perigastric	36	27.561	**<0.001**
Extragastric	15		
**TLNs**			
≤22	26	0.045	0.831
>22	29		
**PLNs** *			
N1	84	159.381	**<0.001**
N2	48		
N3	14		
**NLNs**			
≤9	12	119.545	**<0.001**
>9	54		
**RPDL (%)**			
≤10.0	86	164.430	**<0.001**
10.1–40	47		
>40	13		
**RNPL**			
≤0.18	8	168.730	**<0.001**
0.19–1.70	17		
1.71–7.00	47		
>7.00	81		
**TNM Classification** *			
IIa	75	159.207	**<0.001**
IIb	21		
IIIa	89		
IIIb	48		
IIIc	14		

* According to the 7th UICC TNM Classification of Gastric Cancer.

### Multivariate Survival Analysis

All of the twelve variables above were included in a multivariate Cox proportional hazards model (forward stepwise procedure) to adjust the effects of covariates. In the model, eight block procedures were systematically analyzed to obtain the most intensively independent predicators of the OS of all enrolled patients after curative surgery in accordance with the forward stepwise procedure (likelihood ratio). In the first block analysis, age at surgery, tumor location, tumor size, type of gastrectomy, and Lauren’s classification of primary tumor were included in the procedure. Age at surgery (HR = 1.576; 95%CI 1.201–2.068; *P* = 0.001), and type of gastrectomy (HR = 0.506; 95%CI 0.378–0.679; *P*<0.001) were identified as the significantly independent predicators of the OS of enrolled patients after curative surgery, based on the results of the first procedure. Subsequently, the extent of lymph node metastasis (HR = 1.867; 95%CI 1.387–2.513; *P*<0.001) was demonstrated to have a significant association with the OS of enrolled patients after curative surgery in the second block analysis.

**Figure 1:**
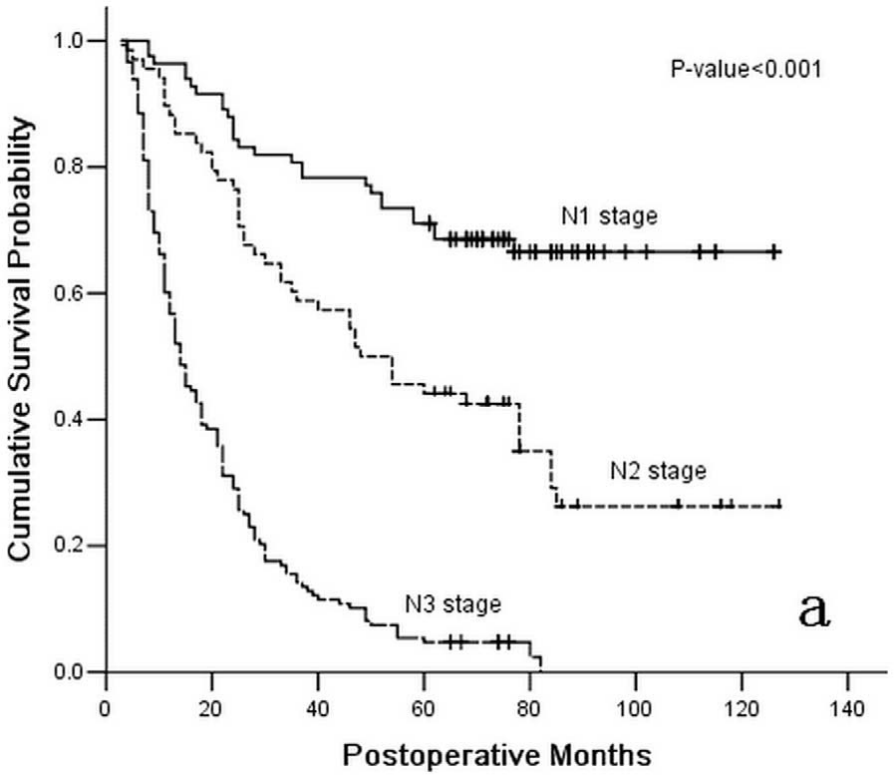
Survival curve for 299 gastric cancer patients with positive lymph nodes following curative resection according to stage subgroup N stage (the 7th UICC TNM Classification of Gastric Cancer).

**Figure 2:**
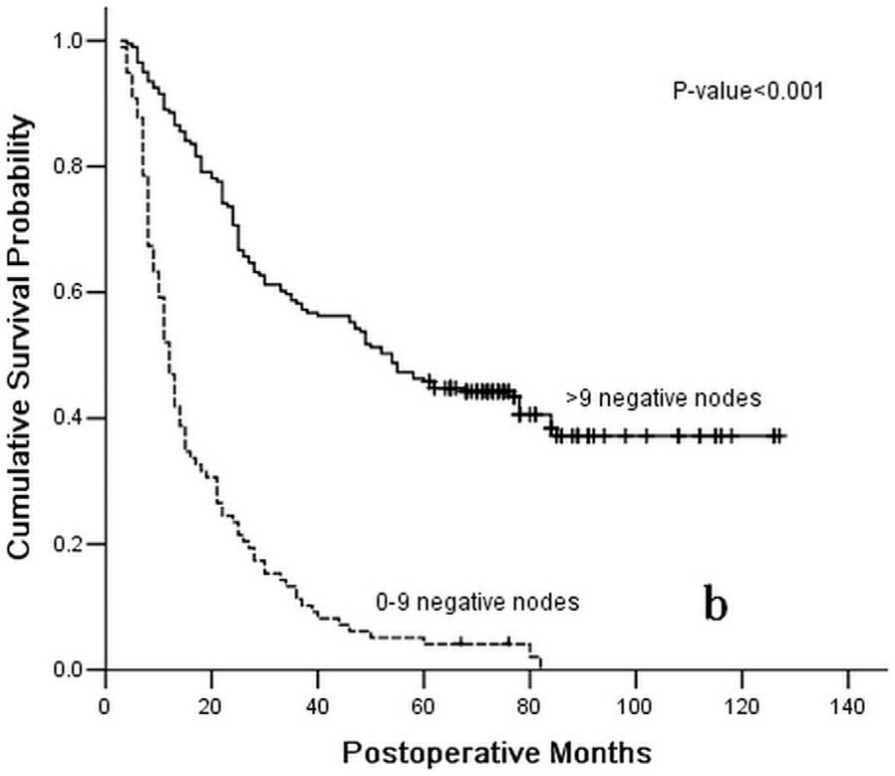
Survival curve for 299 gastric cancer patients with positive lymph nodes following curative resection according to stage subgroup number of negative lymph nodes (≤9, or >9).

**Figure 3:**
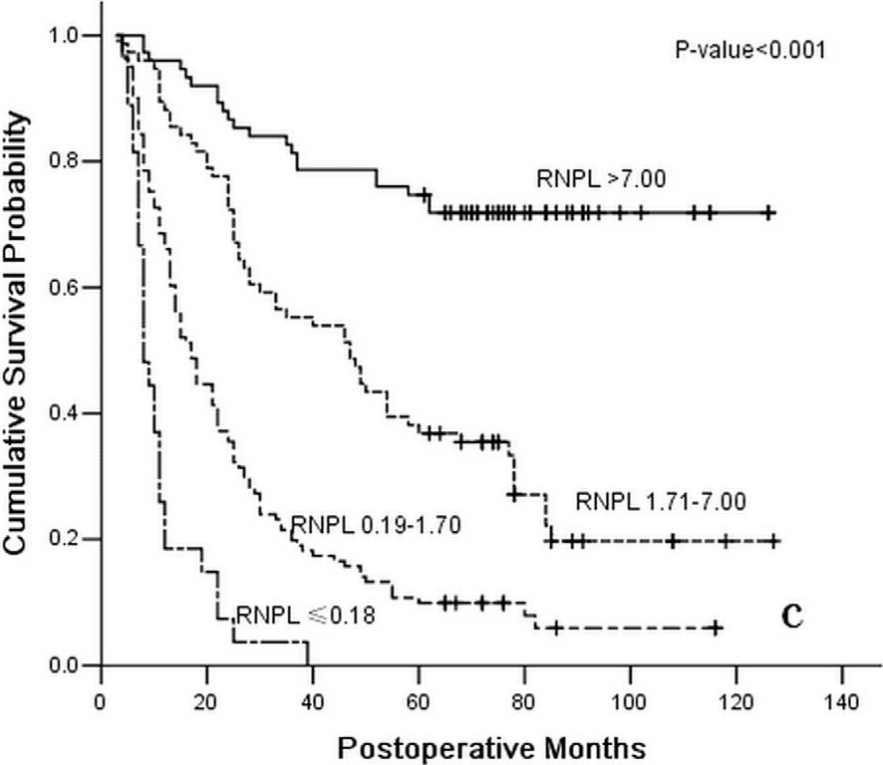
Survival curve for 299 gastric cancer patients with positive lymph nodes following curative resection according to stage subgroup RNPL (≤0.18, 0.19–1.70, 1.71–7.00, or >7.00).

**Table 3:** Multivariate survival analysis of 299 gastric cancer patients with positive node metastasis (according to the Cox proportional hazards model (forward stepwise procedure)).

Block	Variables in the equation	HR	*P* value
1	**Age at surgery**	1.576	**0.001**
1	**Type of gastrectomy**	0.506	**<0.001**
2	**Age at surgery**	1.538	**0.002**
2	**Type of gastrectomy**	0.572	**<0.001**
2	**Extent of lymph node metastasis**	1.867	**<0.001**
3	Age at surgery	1.115	0.443
3	Type of gastrectomy	0.763	0.081
3	Extent of lymph node metastasis	0.983	0.912
3	**PLNs**	3.006	**<0.001**
4	Age at surgery	1.147	0.333
4	Type of gastrectomy	0.779	0.109
4	Extent of lymph node metastasis	1.025	0.877
4	**PLNs**	2.525	**<0.001**
4	**NLNs**	0.642	**0.010**
5 and 6	Age at surgery	1.188	0.226
5 and 6	Type of gastrectomy	0.785	0.121
5 and 6	Extent of lymph node metastasis	1.050	0.759
5 and 6	**PLNs**	1.735	**0.004**
5 and 6	NLNs	0.743	0.101
5 and 6	**RNPL**	0.635	**0.006**
7 and 8	Age at surgery	1.135	0.376
7 and 8	Type of gastrectomy	0.767	0.091
7 and 8	Extent of lymph node metastasis	0.988	0.941
7 and 8	**PLNs**	1.715	**0.005**
7 and 8	**NLNs**	0.689	**0.042**
7 and 8	**RNPL**	0.653	**0.011**
7 and 8	**Depth of primary tumor invasion**	1.552	**0.006**

When the PLNs (according to the 7th UICC TNM Classification N stage) were included in the third block procedure, all three clinicopathological variables lost the statistical significances in the predication of the OS of enrolled patients after surgery. The PLNs (HR = 2.525; 95%CI 1.947–3.276;*P*<0.001) and the NLNs (HR = 0.642; 95%CI 0.458–0.899; *P* = 0.010) were identified as the independent predicators of the postoperatively OS of all patients when the fourth procedure of multivariate analysis procedure was accomplished. After the fifth and the sixth block procedures were executed, another independent predicator of OS, which was shown to have significant association with the postoperatively OS of gastric cancer patients with positive node metastasis, was identified as RNPL (HR = 0.635; 95%CI 0.458–0.880; *P* = 0.006) instead of RPDL (*P* = 0.285).

After the last two block analyses of the Cox proportional hazards model, the depth of primary tumor invasion (HR = 1.552; 95%CI 1.138–2.116; *P* = 0.006) was also identified as an independent predicator of the OS of enrolled patients following the curative surgery. Conversely, the 7th UICC TNM classification of gastric cancer was validated to have no association with the OS of enrolled patients (*P* = 0.713). Ultimately, the depth of the primary tumor invasion was identified as an independent postoperative predicator with the OS of gastric cancer patients, as were the PLNs(HR = 1.715; 95%CI 1.176–2.500; *P* = 0.005), the NLNs (HR = 0.689; 95%CI 0.481–0.987; *P* = 0.042), and the RNPL (HR = 0.653; 95%CI 0.470–0.970; *P* = 0.011) (Figures 1, 2, and 3 and Table 3).

### AIC and BIC Values Performance

AIC and BIC values were executed by using Logistic regression according to the survival status of patients when the follow-up was over. We demonstrated that both AIC value and BIC value of the RNPL were the smallest (AIC value = 79.807, and BIC value = 94.609) in the aforementioned four independently prognostic predicators of gastric cancer which were validated by the multivariate survival analysis (Table 4).

**Table 4:** AIC and BIC values performance for evaluation the best prognostic predicator of the 299 gastric cancer patients with positive node metastasis.

Clinicopathological variables	AIC value	BIC value	-2log likelihood value
Depth of primary tumor invasion	80.963	95.765	72.963
PLNs	87.047	101.849	79.047
NLNs	83.002	97.804	75.002
RNPL	79.807	94.609	71.807

### Bivariate Correlation Analysis

The special correlation between the given category of the nodal metastasis and the postoperative OS of the gastric cancer patients with positive node metastasis needs to be elucidated. Thus, we adopted the bivariate correlation method for the further statistical analysis. Through this method, we demonstrated the significant association of the four categories of nodal metastases with the postoperative OS of gastric cancer patients with positive node metastasis. They are as follows: 1) the PLNs (Pearson correlation value = −0.515, *P*<0.001), 2) the NLNs (Pearson correlation value = 0.448, *P*<0.001), 3) the RNPL (Pearson correlation value = 0.494, *P*<0.001), and 4) the RPDL (Pearson correlation value = −0.596, *P*<0.001). In addition, the TLNs were not proven to have statistical associations with the postoperative OS of gastric cancer patients with positive node metastasis (Pearson correlation value = −0.033, *P* = 0.570; Table 5).

**Table 5:** Bivariate correlation analysis of the special correlationship between the clinicopathological variables associated with nodal metastasis and the postoperative OS of gastric cancer patients with positive nodal metastasis.

Special variables	Pearson correlation value	*P* Value
PLNs	−0.515	**<0.001**
NLNs	0.448	**<0.001**
RPDL	−0.596	**<0.001**
RNPL	0.494	**<0.001**
TLNs	−0.033	0.570

### Partial Correlation Analysis

All of the four nodal metastasis categories above were included in a partial correlation method for evaluation the covariate interaction between the OS of patients with positive node metastasis and the given category of the nodal metastasis. We found several novel results as follows (Table 6): 1) NLNs had no significant impact on the correlation between PLNs and the postoperative OS of gastric cancer patients with positive nodes (Partial correlation value = −0.369, *P*<0.001); 2) PLNs had no significant impact on the correlation between NLNs and the postoperative OS of gastric cancer patients with positive nodes (Partial correlation value = 0.247, *P*<0.001); 3) RPDL had a significant impact on the correlation between PLNs and the postoperative OS of gastric cancer patients with positive nodes (Partial correlation value = −0.015, *P* = 0.801); 4) RPDL had a significant impact on the correlation between NLNs and the postoperative OS of gastric cancer patients with positive nodes (Partial correlation value = −0.015, *P* = 0.792); 5) RNPL had no significant impact on the correlation between the PLNs and the postoperative OS of gastric cancer patients with positive nodes (Partial correlation value = −0.330, *P*<0.001); and 6) RNPL had no significant impact on the correlation between NLNs and the postoperative OS of gastric cancer patients with positive nodes (Partial correlation value = 0.147, *P* = 0.011).

**Table 6:** Partial correlation analysis for evaluation the interaction of covariates on the correlation between the OS of patients with positive nodal metastasis and the special variable associated with nodal metastasis.

Control variables	Correlationship	Correlation value	*P* value
NLNs	Between the PLNs and the postoperative OS of enrolled patients	−0.369	**<0.001**
PLNs	Between the NLNs and the postoperative OS of enrolled patients	0.247	**<0.001**
RPDL	Between the PLNs and the postoperative OS of enrolled patients	−0.015	0.801
RPDL	Between the NLNs and the postoperative OS of enrolled patients	−0.015	0.792
RNPL	Between the PLNs and the postoperative OS of enrolled patients	−0.330	**<0.001**
RNPL	Between the NLNs and the postoperative OS of enrolled patients	0.147	**0.011**

## Discussion

Nodal involvement is one of the most crucial indicators of prognosis of patients with resectable gastric cancer following the curative surgery. Hitherto many categories of lymph node metastasis have been adopted for evaluation the postoperative OS of gastric cancer patients. In brief, three categories of the nodal metastasis have been proposed as the conventional classifications of lymph node metastasis from gastric cancer for predication the prognosis after surgery. They are as follows: the extent of lymph node metastasis, the PLNs, and RPDL.

Generally, the extent of lymph node metastasis cannot be validated as an unfavorable predicator the OS of gastric cancer patients due to the disunion of the criteria of the lymphadenectomy intra-operation worldwide [19]–[22]. It has achieved the consensus that the PLNs should be an intensively prognostic indicator of gastric cancer after curative resection ^7^. However, the Will Rogers phenomenon cannot be avoided if the TLNs are too small to obtain the precise information of nodal metastasis [23], [24]. Tokunaga et al [25] demonstrated that D2 plus para-aortic lymph node dissection (D3 lymphadenectomy according to JGCA) was beneficial to the prognosis of gastric cancer patients with positive para-aortic lymph node metastasis, because of nodes for pathological examination are acquired. Sianesi et al [26] retrospectively reviewed 282 patients who underwent curative resection for gastric cancer at Parma University Hospital between 2000 and 2007. Using the pearson correlation test, they eventually showed that the TLNs were significantly associated with the PLNs (p<0.0001) but not with RPDL. It is so important that the TLNs should be required to achieve the base-line, not less than 15 according to the UICC/AJCC TNM classification for gastric cancer, for acquisition reliable nodal metastatic stage [27].

In addition, many investigators demonstrated the RPDL was the most intensive category of the nodal metastasis for predication the postoperative OS of gastric cancer patients, owing to its inhibition of the stage migration regardless of the number and the extent of lymph node dissection [28]–[30]. Nevertheless, the prognostic superiority of the RPDL has been still controversial for many years [8]. Our previous study demonstrated the RPDL was inferior to the PLNs for predication the OS of gastric cancer patients with 15 or more TLNs after curative resection by analyzing with the control-case match method [12]. Bilici et al [31] reported both the RPDL and the 5th UICC/AJCC pN stage of gastric cancer were detected as independent prognostic factor by multivariate analysis. Unfortunately, no significant prognostic superiorities of the RPDL were shown in that investigation, comparing to the 5th UICC/AJCC pN stage. Although it might help to stratify patients in terms of prognosis when the TLNs are limited, Kulig et al [32] demonstrated that the RPDL couldn’t be regarded as a standard category of lymph node metastasis alternative to other categories after curative gastrectomy plus the extended lymphadenectomy.

The UICC/AJCC TNM classification for gastric cancer is a kind of manual containing the periodical promotion and modification. The 7th UICC/AJCC pN stage of gastric cancer is the latest edition for evaluation the positive node metastasis from gastric cancer, which has been validated to be more accurate than the previous edition of the pN stage for predication the OS of patients after surgery [33]–[35]. Actually, comparatively elaborate pN stage of the 7th UICC/AJCC TNM classification for gastric cancer can significantly improve the prognostic precision of patients following the curative resection. In our previous study, we demonstrated the 7th UICC/AJCC pN stage of gastric cancer was superior to the 5th/6th UICC/AJCC pN stage or the RPDL for predication the OS of gastric cancer patients with 15 or more TLNs after curative resection [36].

Theoretically, NLNs may be associated with two important conditions to affect the prognosis of gastric cancer patients after curative resection as follows: 1) immune condition against the malignant disease; and 2) micro-metastasis from the primary tumor. In the previous investigation, we found that the NLNs could enhance the postoperatively prognostic predication of the RPDL to gastric cancer patients [13]. Through an elaborately subgroup analysis of the clinicopathological data of patients, we demonstrated that the subgroups of gastric cancer patients in accordance with the same RPDL may have significantly different postoperative OS owing to the nonconformity of NLNs [13]. Huang et al [37] reviewed the clinicopathological data 634 gastric cancer patients who underwent a curative resection (R0) of lymph nodes with distal gastrectomy from 1995 to 2004. They confirmed that increasing the negative lymph node count could reduce the RPDL and improve the survival rate of gastric cancer patients. Similarly, we previously demonstrated that the harvest of enough negative lymph nodes was the most important factors to improve the OS of gastric cancer patients with perigastric node metastasis after curative gastrectomy plus D2 lymphadenectomy [14].

The TLNs are composed of positive nodes and negative nodes simultaneously. The sum of the number of positive nodes and the number of negative nodes can not be sure to have a close association with the OS of patients, which induces some adverse effects of the RPDL for predication the OS of gastric cancer patients after surgery, compared with the RNPL which is straight ratio between negative and positive lymph nodes. Whether or not the RPDL is the most authentic category of the lymph node metastasis for predication the OS of gastric cancer patients remains controversial. Unfortunately, the detailed defects of the RPDL for predication the OS of gastric cancer have not been clarified. In this study, we demonstrated that the RNPL is more suitable for predication the prognosis of gastric cancer patients with nodal metastasis than other factors. We believe that, compared with RPDL, the RNPL could yield more accurate statistical results by providing direct and comprehensive information on nodal metastasis, micro-metastasis, and immune conditions against the malignant disease of patients. Although our present study is only a retrospectively small-scale investigation, we obtained information that could be beneficial for selection a more suitable category of nodal metastasis than RPDL for predication the OS of gastric cancer patients after curative resection.
